# EGCG in Green Tea Induces Aggregation of HMGB1 Protein through Large Conformational Changes with Polarized Charge Redistribution

**DOI:** 10.1038/srep22128

**Published:** 2016-02-22

**Authors:** Xuan-Yu Meng, Baoyu Li, Shengtang Liu, Hongsuk Kang, Lin Zhao, Ruhong Zhou

**Affiliations:** 1 Institute of Quantitative Biology and Medicine, SRMP and RAD-X, Collaborative Innovation Center of Radiation Medicine of Jiangsu Higher Education Institutions, Soochow University, Suzhou 215123, China; 2 IBM Thomas J. Watson Research Center, Yorktown Heights, NY 10598, USA; 3 Department of Chemistry, Columbia University, New York, NY 10027, USA; 4 Department of Physiology and Biophysics, Virginia Commonwealth University, School of Medicine, Richmond, Virginia, VA, USA

## Abstract

As a major effective component in green tea, (−)-epigallocatechin-3-gallate (EGCG)’s potential benefits to human health have been widely investigated. Recent experimental evidences indicate that EGCG can induce the aggregation of HMGB1 protein, a late mediator of inflammation, which subsequently stimulates the autophagic degradation and thus provides protection from lethal endotoxemia and sepsis. In this study, we use molecular dynamics (MD) simulations to explore the underlying molecular mechanism of this aggregation of HMGB1 facilitated by EGCG. Our simulation results reveal that EGCG firmly binds to HMGB1 near Cys106, which supports previous preliminary experimental evidence. A large HMGB1 conformational change is observed, where Box A and Box B, two homogenous domains of HMGB1, are repositioned and packed together by EGCG. This new HMGB1 conformation has large molecular polarity and distinctive electrostatic potential surface. We suggest that the highly polarized charge distribution leads to the aggregation of HMGB1, which differs from the previous hypothesis that two HMGB1 monomers are linked by the dimer of EGCG. Possible aggregating modes have also been investigated with potential of mean force (PMF) calculations. Finally, we conclude that the conformation induced by EGCG is more aggregation-prone with higher binding free energies as compared to those without EGCG.

High-mobility group 1 protein (HMGB1) is a bi-functional protein that functions as chromatin-associated proteins to regulate transcription in the nucleus, and it can also be released extracellularly to mediate the response to inflammatory stimuli like infection and injury^1^. Structurally HMGB1 contains two homogenous DNA-binding domains Box A and Box B connected by a 20-amino-acid-long loop, followed by an acid tail (AT) rich in GLU and ASP residues. Early NMR studies attained the complex structures of single box combined with DNA, indicating that both boxes are capable of independently binding to DNA^2^.

The inflammatory role of HMGB1 has been widely studied, for example, Wang *et al.*^3^ revealed back in 1999 that sepsis (a systemic inflammatory response syndrome) is closely related to HMGB1 released by the activated microphages in serum. Excessive accumulation of HMGB1 may lead to pathogenesis of lethal endotoxemia and sepsis^2^,^4^. As HMGB1 concentration increases in serum 12–18 hours later than early cytokines (such as TNF and IL-1), it has been recognized as an effective therapeutic target in sepsis^1^,^3^.

(−)-Epigallocatechin-3-gallate (EGCG) is the major effective component of green tea, and it is associated with many health benefits against multiple diseases including inflammation and rheumatoid arthritis^5^. Li *et al.* has shown that the anti-inflammation effect can be attributed to the inhibition of endotoxin-induced HMGB1 release^6^. It has been elucidated in recent experiments that EGCG induces the aggregation of HMGB1 and subsequently stimulates the autophagic degradation in the macrophages^6^,^7^, while the underlying molecular mechanisms remain elusive.

In addition to anti-inflammation, abundant evidences have shown that green tea consumption significantly reduces cancer risks, and consequently, EGCG, its major effective component, has emerged as a potential promising candidate for anti-cancer agents^8^,^9^,^10^,^11^,^12^. HMGB1 is also a protein implicated in cancer malignant development by stimulating migration and enhancing proliferation. Patients suffered from various cancers are noted with elevated levels of HMGB1 in blood^13^. However, it remains inconclusive whether EGCG as an anti-cancer agent is related to the HMGB1 protein. The influence of EGCG on the aggregation of HMGB1 could be a route for its anti-cancer efficacy.

Thus, it is important to understand the interaction between EGCG and HMGB1, which not only provides novel insights into the biological function of EGCG, but also promotes its wider applications. As mentioned above, despite extensive studies, the underlying molecular mechanism on how EGCG facilitates theHMGB1 aggregation is still unclear. Previously, Western blotting indicated that in the presence of EGCG, HMGB1 can accumulate in different aggregating degrees (dimer, trimer or even higher oligomer of proteins). The aggregation is inhibited by DTT (a reducing agent specific to S-S or S-O bond for the Cys residue), which confirms that a S-O bond between Cys and EGCG is a critical factor to the aggregation of HMGB1. It was proposed that theasinensin, a dimer of EGCG forming by auto-oxidization, may be another factor to the HMGB1 aggregation^7^. However, whether one the asinensin connecting to two HMGB1s leads to the dimerization of proteins is still inconclusive. Also, the bridge role played by theasinensin cannot explain the high-order aggregation of HMGB1 observed in the Western blot.

In the present study, we utilize molecular dynamics (MD) simulations combined with potential of mean force (PMF) calculations, which are both widely used in biomolecular simulations^14^,^15^,^16^,^17^,^18^,^19^,^20^, to investigate the molecular mechanism by which EGCG induces the aggregation of HMGB1. Our study aims to show how EGCG mediates HMGB1 protein aggregation through a very unique conformation with large molecular polarity. Also, we investigate how the highly polarized structure of HMGB1 with positive-charged ‘head’ and negative-charged ‘tail’ affects the formation of oligomer.

## Methods

### Model refinement

Among several solution NMR structures of HMGB1 protein available in the PDB database, we selected the one with the PDB code 2YRQ because it is the only structure that contains both Box A and Box B. The missing acidic tail (AT, from K167 to E215) in the 2YRQ was reconstructed using the Loop Refinement (MODELER) module in the Discovery Studio software (Accelrys Inc., San Diego, CA, USA). For each model involved in this study, a total of five loop structures were generated and optimized by using simulated annealing simulations. The DOPE (Discrete Optimized Protein Energy) score was used for evaluating the quality of the models^21^. The best scored loop structure was selected to constitute the complete model of proteins.

### Molecular dynamics simulations

The protein was solvated with SPC water molecules and its excess negative charge was neutralized by Na^+^. GROMACS v4.6.5^22^ was used to conduct the simulation with the GROMOS96 53a6 force field^23^. The parameters for EGCG were adopted from a previous study in Sun’s Lab^24^. Long range electrostatics were calculated using the particle mesh Ewald (PME) method^25^ with a 12 Å cut-off. Van der Waal interactions were modeled using Lennard-Jones 6–12 potentials with a 14 Å cut-off. All production runs were performed with the NPT ensemble, with a constant temperature at 300 K using the Berendsen thermostats, and a constant isotropic pressure at 1 bar using the Berendsen barostats^26^. All bonds were constrained with the LINCS algorithm^27^. The time step was set to 2 fs and the neighboring list was updated at every 10 time steps.

Prior to production runs, following similar protocols in our previous studies^28^,^29^,^30^,^31^,^32^,^33^, energy minimization of 3000 steps with steepest descent method were carried out on each system followed by a 0.1 ns equilibration process in which position restrain was applied to the backbone of the protein and EGCG molecule using a constant force of 1000 kJmol^−1^ nm^−2^. Production runs with lengths of 100–300 ns were conducted and coordinates were saved every10 ps for analysis. VMD^34^ and Chimera^35^ programs were used for visualization. Electrostatics potential of proteins were calculated using DelPhi module in Discovery Studio software (Accelrys Inc., San Diego, CA, USA) with default parameters (grid points per axis is 65 and 50% grids is filled by solute; the dielectric constant of the solvent and solute interior is 80 and 2, respectively; ionic strength 0.145 mol L^−1^ was used).

### PMF simulations

Pulling simulations were conducted on the dimer system to generate the starting configurations for umbrella sampling. The distance between the center of mass (COM) of the protomers was used as a reaction coordinate. The dimer interface was carefully defined based on coordinates of three selected interfacial atoms. For m17 Z system, three atoms are Cα of R10, Cα of S15 and Cα of K43; for m14 head system: Cα of K30, Cα of G83 and Cα of E194. The pulling force applied to the COM of one protomer was along the normal vector of the defined interface with a constant of 1000 kJ mol^−1^ nm^−2^ and a pulling speed of 0.01 nm ps^−1^. For m17 X system, the pulling force was along X axis because its dimer interface was basically parallel to the YZ plain (the pulling constant and pulling speed are the same). The final distance between the COM of protomers reached at 9–10 nm.

Umbrella sampling was used to calculate the potential of mean force (PMF) that measures the relative strength of interaction between the dimer interfaces. The umbrella windows were generated using 0.1 nm spacing along COM distance. Therefore totally 48, 72 and 45 windows were generated for m17 Z, m17 X and m14 head systems, respectively. For each window of the umbrella sampling, the system was conducted 10 ns MD simulations after equilibration. Both equilibration and production runs were under NPT ensemble. A total of 1.65 μs of simulation time was conducted to investigate the interfaces in three systems. Analysis of results was performed with the weighted histogram analysis method (WHAM)^36^.

## Results and Discussion

### Binding of EGCG on HMGB1 requires participation of both Box A and Box B

**Figure 1:**
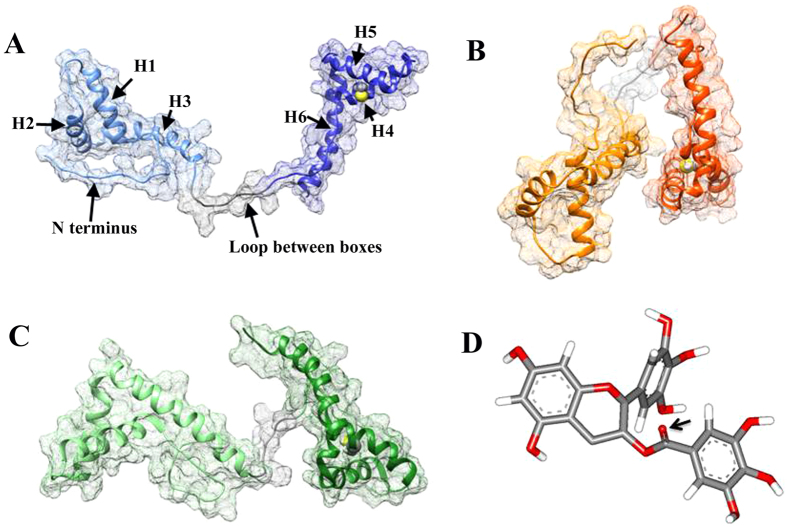
(**A**–**C**) Representative NMR structures of HMGB1 (PDB: 2YRQ) and (**D**) EGCG molecule. Each box of HMGB1 contains three helices, and nomenclature denoted in panel (**A**) (Box A: H1, H2 and H3, and Box B: H4, H5 and H6). The carbonyl O atom that has highest chemical activity is denoted by an arrow in panel (**D**). The reconstructed AT region was not in the NMR structure, thus not shown.

The aggregation of HMGB1 in the presence of EGCG was abrogated by applying DTT *in vitro*. This observation implies that a S-O bond between the Cys residue and EGCG plays a critical role in the aggregation process. HMGB1 has three Cys residues in total: Cys23, Cys45 and Cys106. Cys23 and Cys45, which are located in Box A, form a disulfide bond in the mild oxidative condition^37^ and cannot involve in forming S-O bond with EGCG; thus Cys106 in Box B is more likely to interact with EGCG. For EGCG, carbonyl O has higher chemical reactivity compared with the O atoms in the phenol hydroxyl groups (Fig. 1D). Therefore, we first hypothesize that the S-O bond may be formed between Cys106 of HMGB1 and carbonyl O of EGCG.

**Figure 2:**
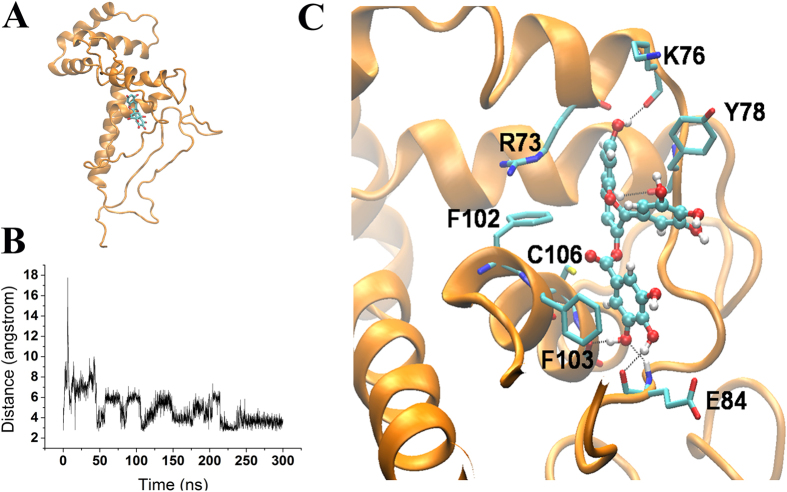
(**A**) Complex structure of HMGB1-EGCG. (**B**) Distance of carbonyl O of EGCG from the S atom of Cys106 as a function of time. The carbonyl oxygen keeps its distance at around 3.5–6 Å from the S atom of Cys106 throughout the whole simulation time. (**C**) EGCG is stabilized at the binding site through forming five hydrogen bonds and π-π stacking with Y78 and F103, respectively.

NMR data (PDB code 2YQR) suggest 20 different structures of HMGB1. The conformational difference mainly arises from the long flexible loop that connects both Box A and Box B, which alters the relative position of the boxes. Since Cys106 in Box B mainly participates in binding to EGCG, we initially built the simulation system containing only Box B and EGCG with its carbonyl O approaching the S of Cys106. We tried several different configurations of EGCG and Box B, however, EGCG was never stably bound to the binding site of Box B in these simulations. This observation implies that Box A might also play a role in EGCG binding. We thus carefully inspected the 20 structures of HMGB1 and screened them using the following criteria: 1) disregard the structures where Box A has no contact with Box B (Fig. 1A); 2) discard the structures where two domains are too close to accommodate EGCG; 3) take the structures that residues from Box A might have contribution to EGCG binding (namely, the structures are preserved if their one or more residues from Box A are spatially close to the C106 and consist of the putative EGCG binding site). As a result, three structures, m7, m10, and m17, satisfying these criteria were considered as candidates for further investigation. We manually positioned EGCG in proximity to the binding site with the EGCG carbonyl O approaching the S of Cys106. After over hundred nanoseconds of MD simulations, EGCG and m17 formed a stable complex conformation with the carbonyl O of EGCG 3.5–6 Å away from the S atom of Cys106.

It turns out that the binding of EGCG to the HMGB1 is very stable. EGCG forms five hydrogen bonds with four residues, K76, Y78, E84 (two H-bonds) and S107, respectively, and has two pairs of π-π stacking which are epigallo ring packed with Y78 and 3-gallate ring with F103 (Fig. 2). The interactions between EGCG and K76, Y78, E84also contribute to the stabilization of the conformation of the loop connecting Box A and Box B. Moreover, EGCG drives the conformational change of the protein as well, especially by altering the interactions between the helices. We observed a stable contact between the phenyl ring of F102 (in the H4; see Fig. 1A) and guanidyl group of R73 (in the H3). The pair was surrounded by a series of hydrophobic residues (F105 and Y109in the H4, L129 and W133 in the H5) as well as the catechin ring of EGCG. These residues together with catechin ring constitute a hydrophobic core buried inside the protein, leaving the hydrophilic residues, especially the charged ones in the helices exposed in the solution.

Such EGCG-induced conformational change significantly changed the electrostatic potential surface of the protein which will be discussed in details in the next section. Additionally, note that the bending in the middle of H3 was aggravated in the presence of EGCG. The H3 was bent at position D67 in all 20 NMR structure of HMGB1 with different bending degrees (Fig. S1). The presence of EGCG almost broke down the helix at D67 position, which was not observed in the simulations without EGCG.

### Unique electrostatic surface of HMGB1 induced by EGCG leads to the protein aggregation

**Figure 3:**
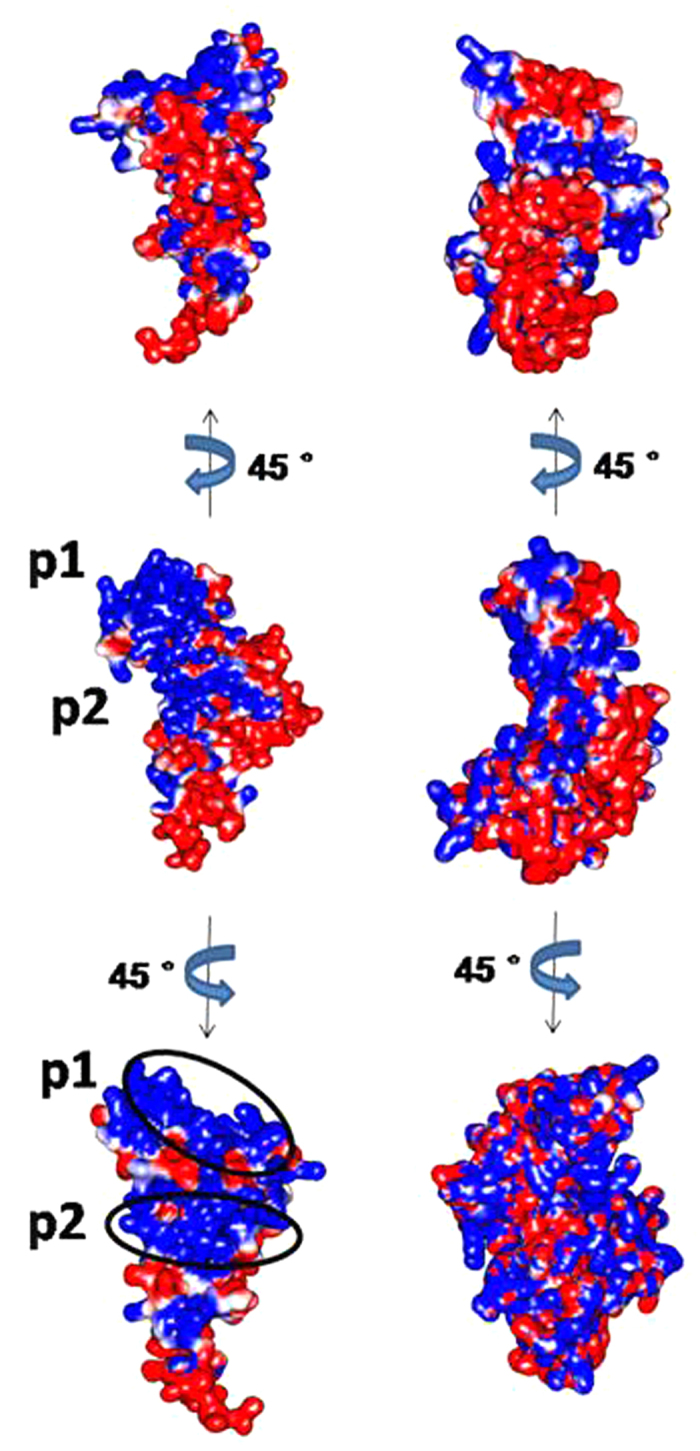
Electrostatic potential surface of m17 in the presence of EGCG (left panel) and the absence of EGCG (right panel). p1 and p2, positive parts of surface in m17 are circled.

HMGB1 protein carries many charged residues including 43 lysines, 8 arginines, 36 glutamates and 20 aspartic acids, which constitutes 50% of the total residues. Therefore, it is critical to examine the distribution of charged residues under different HMGB1 conformations to understand the role of its unique electrostatic properties during the EGCG-induced dimerization. We generated the electrostatic surface potential (ESP) of the protein by the DelPhi program embedded in Discovery Studio. Surprisingly, the protein ESP was significantly altered by EGCG. Indeed, HMGB1 in the presence of EGCG showed a very unique ESP that a ‘head’ of the protein (contributed by Box A and part of Box B) has positively charged surface while a ‘tail’ (contributed by acidic tail) has negatively charged one. This indicates that the HMGB1 protein is likely to form a ‘head-to-tail’ dimer with one monomer stacking up-side down to the other. As long as the highly polarized ESP does not change during protein aggregation, higher multimers may form based on this growing pattern (more discussions in the next section on simulations of dimerization). The positive surface can be subdivided into two regions, termed p1 and p2 (Fig. 3). The region p1 was contributed by the N-terminus, H1 and H2 from Box A whereas p2 by H4 and H5 from Box B, and part of H3 from Box A. Both p1 and p2 were linked by K65 located on H3.

As for comparison, we also conducted 200 ns-long MD simulations on the m17 in the absence of EGCG (control run), of which charges distributed more or less evenly on the protein surface (Fig. 3). The m14 structure, a representative of the extended protein conformation, also exhibited even distribution of charges at the molecular surface after 200 ns simulation (Fig. S2).

We also calculated the dipole moment (μ) which measures the net molecular polarity. The average dipole moment was calculated based on the last 50 ns trajectory of each system. It is quite clear that, with EGCG-binding, the polarity of HMGB1 increased by more than twice as compared with m17/m14 in the absence of EGCG (Table 1). The strong dipole moment induced by EGCG along with the unique polar electrostatic surface probably benefits the aggregation of the protein (more below).

### Simulations of HMGB1 dimerization

Since m17 in the presence of EGCG has the highest potency to aggregate, we chose the m17+EGCG as our representative monomer system for the aggregation simulations. As shown in Fig. S3, three simulation systems were setup as following: 1) Translating a monomer by 5 nm along the X axis to generate at least 1 nm minimal distance between the monomers (termed X system); 2) translating a monomer by 5 nm along the Z axis to generate at least 1 nm minimal distance between the monomers (termed Z system); 3) rotating the system by 180 degree along the Y axis followed by a translation to generate at least 1 nm minimal distance between the monomers (termed Y system). We prepared three different initial configurations to investigate the dependence of the dimerization on the orientations of monomers. The Z system corresponds to an ideal conformation for the dimerization whereas both the X and Y systems represent the situation where the dimerization is less likely to occur. By comparison among these systems, we may find the correlation between the dimerization and the orientation of monomers. Each system was simulated for 200 ns without any restraints on the proteins and EGCG.

**Figure 4:**
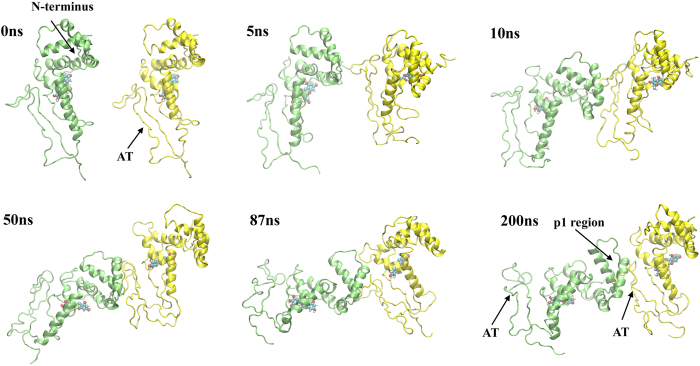
Dimerization of Z system (m17+EGCG). The protomer A is colored in green and protomer B in yellow. The N-terminal, AT (acidic tail) and p1 regions are denoted by arrows.

As aforementioned, with EGCG, m17 shows highly polarized surface with Box A having a positive surface while the AT having a negative one. Thus, it is predicted that Box A of one protomer is most likely to face towards the AT of the other driven by the electrostatic attraction when two HMGB1s approach each other. The Z system was designed to resemble such situation. As expected, the N-terminal of protomer A interacted with the AT of protomer B in a short simulation time (with in 5 ns; Fig. 4). Then the contacting interface was expanded to the entire p1 region, and remained stably until the end of simulations. The RMSD of the dimer was also saturated after10 ns. While the N-terminal of protomer A steadily interacted with the AT of protomer B, the AT of protomer A was still free and fully exposed in the solution to attract next monomer to form even higher oligomers.

**Figure 5:**
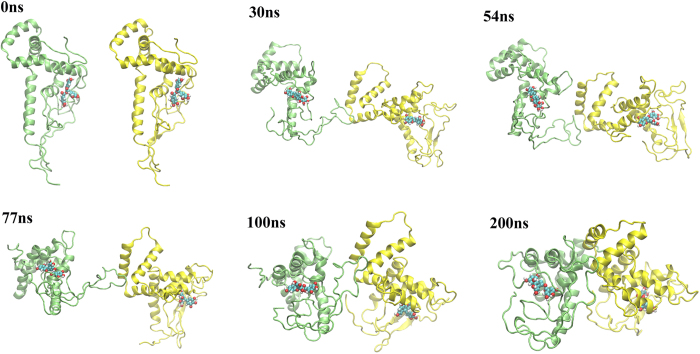
Dimerization of X system. The protomer A is colored in green and protomer B in yellow.(m17+EGCG).

In the X system, the p1 regions and AT domains from two protomers kept in parallel, respectively (Fig. S3). After 30 ns of simulation, the AT of protomer A interacted with the p1 of protomer B as observed in the Z system (Fig. 5; 30 ns). After that, the AT of protomer A began to be transiently detached from the boxes of the protein as it interacted only with the p1 domain of protomer B (Fig. 5; 77 ns). Meanwhile, because of the lack of interaction with the AT, both Box A and Box B of protomer A were inclined to collapse towards the core. At 100 ns, the AT of protomer B also interacted with the H6 of the protomer A so that neither of ATs were free, and thus none of them can interact with a third HMGB1. Moreover, the dimerization process remarkably decreases dipole moment of each protomer whose reduction hinders promoter’s ability to attract any third HMGB1 molecule (Table 1). Therefore, we may conclude that the formation of the dimer suppresses further oligomerization and aggregation processes with this X form initial conformation.

In the Y system, a quick collision occurred between the molecules without any changes of relative orientation compared with their initial orientation (Fig. S4). And the system remained the same state throughout the simulation. This represents a rare but kind “ideal” situation for the dimerization, however, this Y-system represents a so-called “closed-form” dimerization^38^, which will not prompt further elongation, thus not useful for our current discussion. Similar closed-form dimerization was also found in a previous study on human γ-D crystalline unfolding through a domain swapping mechanism^38^. Since in this study, we are mainly focused on how open-form dimerization was initiated by EGCG, this closed-form will thus not be further discussed in next sections.

To investigate the possibility of aggregation in the absence of EGCG, we also ran the simulations with m14 alone (as control runs), which has smaller dipole moment than m17+EGCG (Table 1). It has a belt of negatively charged region contributed by the AT, and positively charged ones in both Box A (head) and Box B (tail) (Fig. S2). Hence, we prepared three different initial configurations for MD simulations: 1) the head of protomer A pointed to the AT of protomer B, 2) the tail of protomer A pointed to the AT of protomer B, and 3) two protomers stayed in parallel along X axis.

**Figure 6:**
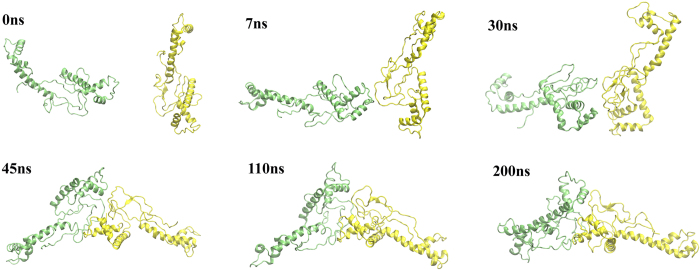
Dimerization of head-to-AT system (m14).

In the head-to-AT simulations, the protomer A approached to the protomer B maintaining its initial orientation. Apparently, the head of protomer A interacted with the AT of protomer Bat early stage (Fig. 6; 7 ns).However, it turns out that the head-to-AT interaction is transient. As the system was evolved, stable interfacial contacts were finally established between the AT and the loops of the proteins (N-terminal of H2 — AT, AT — AT, AT — N-terminal, and AT — the loop between boxes). On the other hand, in the tail-to-AT system (Fig. S5), Box B did not interact with the AT even though they were set to be close to each other in the initial configuration. Instead, it began to interact with the other Box B at 56 ns and the two Box Bs hooked each other at the end of simulation. In the parallel system, the interface between the protomers kept changing during the simulation (Fig. S6). No stable interface was formed by the protomers even when we extended the simulation time to 300 ns. This result implies interaction between the protomers is unfavorable in the absence of EGCG.

Taking all the above six trajectories together, we have three important observations: 1) in the absence of EGCG, for all the conformations we have tested, only the head-to-AT conformation showed some dimerization; 2) with EGCG, protomers of m17 started to interact with each other much earlier than those of m14, and 3) the interfaces between m17 and EGCG were more stable than those of m14. Both 2) and 3) make m17 more prone to EGCG-induced aggregation.

**Figure 7:**
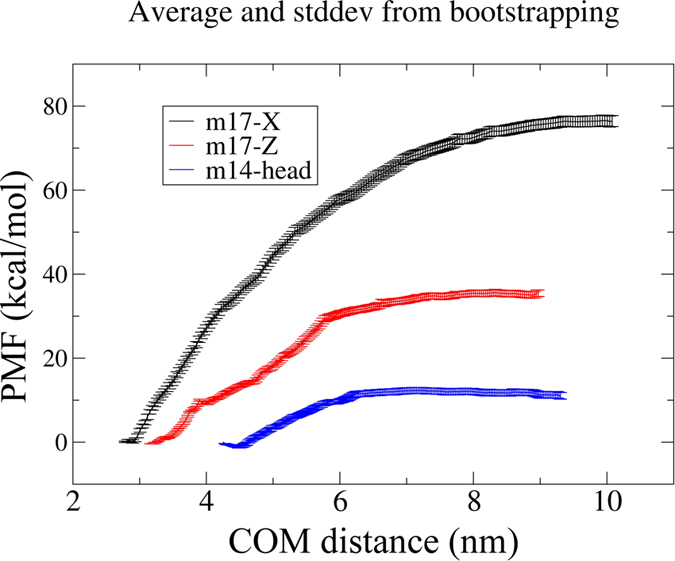
PMF calculations of three representative simulations with an estimation of error bars. Statistical errors were estimated with bootstrap analysis^46^.

In order to further measure the relative strength of the HMGB1 dimers (starting from m17 or m14, with or without EGCG), we performed the PMF calculations combined with umbrella sampling using the simulated dimer configuration as a starting point. Among the three simulations of m14, the head-to-AT was selected as a representative one because it formed stable interface (compared with parallel system) and had the largest buried surface area (compared with tail-to-AT system). Regarding the m17+EGCG, both X and Z systems were chosen, which represent possible closed and open dimer conformations, respectively. PMF calculations for the three systems clearly show that the binding strength is in the order of X (m17+EGCG) > Z (m17+EGCG)>head-to-AT (m14) (see Fig. 7 and Fig. S7). The PMF values of the m17+EGCG X and Z systems were about 7 and 3 times that of the m14 system, respectively, indicating that binding energy of monomer-monomer interaction is much higher in the presence of EGCG. It should be noted that the PMF values here, particularly for m17+EGCG X (77.6 kcal/mol) and Z (35.9 kcal/mol) systems, might seem too large as compared to normal protein-protein interactions. This might be related to three unique and important factors: 1) ~50% of residues in HMGB1 are charged, which is very unusual and unique; 2) the EGCG-induced bipolar distribution of charges can result in large dipole moments and thus significant electrostatic interactions between aggregating monomers; and 3) our current umbrella sampling might not fully incorporate the very large protein flexibility during the mechanical-forced separation of the dimers, which should in theory lower the energy barrier for separation somewhat (in other words, the binding energies from the current PMF calculations are upper bounds). In general, HMGB1 has large flexibility due to the long loops between the Boxes and the bending of H3 (Fig. S1). Such flexibility may cause significant conformational changes during separation (Fig. S8), for example, in the X system of m17+EGCG, the N-terminus can bring additional flexibility that can decrease the separation free energy barrier somewhat once the full conformational changes can be included. In addition, a previous similar study on the assembly of a 9-residue amyloid peptide (GAV-9) on mica shows a ~32 kcal/mol separation free energy barrier along its hydrophobic edge^39^, so the current binding energy values might not be totally out of scope. Nevertheless, these PMF calculations confirm the observations in above MD simulations, with a noticeable trend of X (m17+EGCG) > Z (m17+EGCG)>head-to-AT (m14) in terms of HMGB1 dimerization preference.

It should be noted that the protein-protein interface often play a significant role during protein dimerization or aggregation^14^,^15^,^28^,^40^. These interfaces are often dry, which provide enormous hydrophobic forces for aggregation, as shown in many of our previous studies as well^14^,^28^,^41^. However, surprisingly, in this HMGB1 dimerization, the protein interfaces were all found to be wet during the dimerization, as illustrated in Fig. S9 as one example. This is largely due to the extremely polar interfaces HMGB1 imposes, where salt-bridges are either formed directly or facilitated through bridging water molecules.

## Conclusions

Recent *in vitro* and *in vivo* experimental evidences indicate that EGCG can induce the aggregation of HMGB1 protein by binding to HMGB1, which subsequently protects the host from sepsis and endotoxemia^6^,^7^. Here we conducted multiple MD simulations and PMF calculations to illustrate a possible novel mechanism for this phenomenon. Our MD simulations confirmed that EGCG can stably bind to the Cys106 site and then induce the conformational change of the protein. The induced conformation has large molecular polarity (i.e. large molecular dipole moment), thus it tends to aggregate together through favorable electrostatic interactions. The PMF calculations also support the idea that the induced conformation has higher propensity for the aggregation as compared to the apo conformations of protein without EGCG.

It should be pointed out that EGCG was also shown to inhibit amyloid peptide aggregation by remodeling amyloid fibrils, changing the aggregating pathway of polypeptides, and converting the assembly into small and less toxic aggregates^42^,^43^,^44^,^45^. This seemingly opposite role of EGCG (as compared to the current case) was also suggested for potential treatments of amyloid diseases, such as Alzheimer’s, Parkinson’s diseases^42^,^43^,^44^,^45^. Our current study, on the other hand, shows that EGCG can facilitate the aggregation of HMGB1 by changing its conformation and strongly altering its molecular properties. Therefore, EGCG can function in potentially opposite ways: either promoting the disaggregation of amyloid fibrils, or facilitating the aggregation of HMGB1 through unique reorganization of the protein conformation. This apparent contradiction mainly arises from the fact that there are large, multi-faced, molecular morphological/conformational changes induced by EGCG. EGCG has multiple functional groups capable of forming multiple hydrogen bonds, hydrophobic and π-π interactions simultaneously, which promote interactions with more than one binding sites in the peptides and proteins. In the case of HMGB1, EGCG acts like a glue that ties Box A and Box B together. Whether other EGCG targets such as amyloid peptides aggregates/oligomers utilize similar molecular mechanism or not is an interesting question that deserves further investigation.

## Additional Information

**How to cite this article**: Meng, X.-Y. *et al.* EGCG in Green Tea Induces Aggregation of HMGB1 Protein through Large Conformational Changes with Polarized Charge Redistribution. *Sci. Rep.* **6**, 22128; doi: 10.1038/srep22128 (2016).

## Supplementary Material

Supplementary Figures

## Figures and Tables

**Table 1:** Dipole moment of HMGB1 in the simulated systems (for Dimer systems, see the main text for the definition of X5, Y180 and Z5 etc).

Simulated Systems	Monomer			Dimer (protomer A, protomer B)		
	m17+EGCG	m17	m14	X5	Y180	Z5
μ (Debye)	2881.8	1495.0	1269.4	1714.9, 1759.8	1930.0, 2125.6	2581.4, 2496.2
