# Supplementary material for: EGCG in Green Tea Induces Aggregation of HMGB1 Protein through Large Conformational Changes with Polarized Charge Redistribution
Source: Sci Rep. 2016 Feb 22;6:22128. doi: 10.1038/srep22128 (PMC4762017; doi:10.1038/srep22128)
Supplement: Supplementary Figures [file srep22128-s1.doc]

**EGCG in Green Tea Induces Aggregation of HMGB1 Protein through Large Conformational Changes with Polarized Charge Redistribution**

Xuan-Yu Meng^1,4^,Baoyu Li^1^, Shengtang Liu^1^, Hongsuk Kang^2^, Lin Zhao^1^, Ruhong Zhou^*1,2,3^

*^1^Institute of Quantitative Biology and Medicine, SRMP and RAD-X, Collaborative Innovation Center of Radiation Medicine of Jiangsu Higher Education Institutions, Soochow University, Suzhou 215123, China*

*^2^ IBM Thomas J. Watson Research Center, Yorktown Heights, NY 10598, USA*

*^3^ Department of Chemistry, Columbia University, New York, NY 10027, USA*

*^4^Department of Physiology and Biophysics, Virginia Commonwealth University, School of Medicine, Richmond, Virginia.*

**Supplementary Figures**


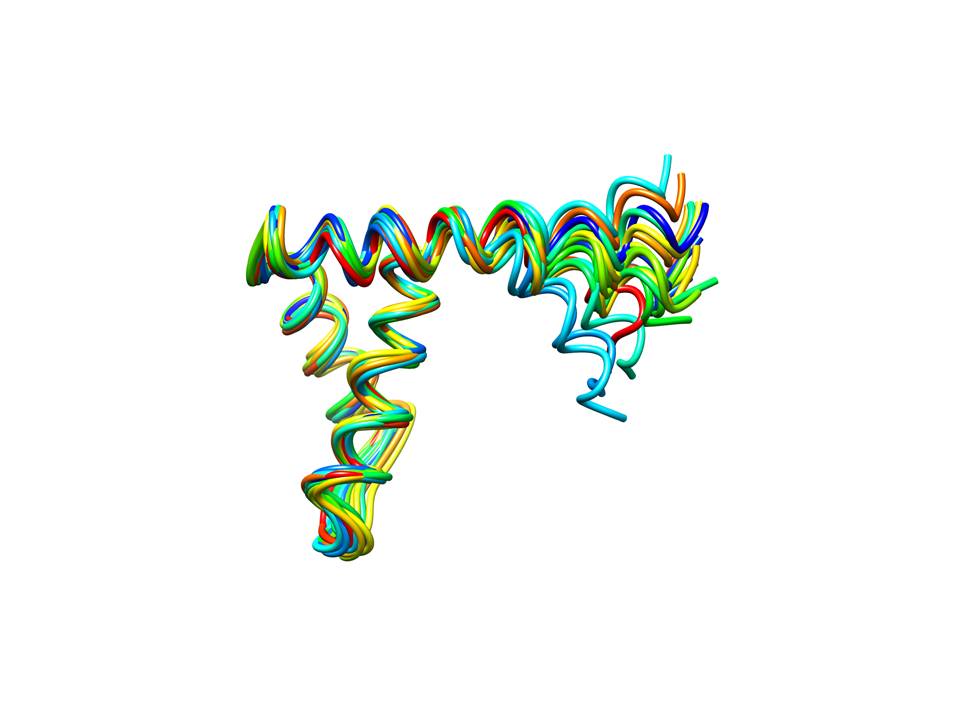


**Fig. S1.** Superposition of Box A of 20 NMR structures indicates the bending of H3 at position D67. The Bending of H3 together with the long loop between Boxes contributes to the flexibility of the HMGB1 protein.


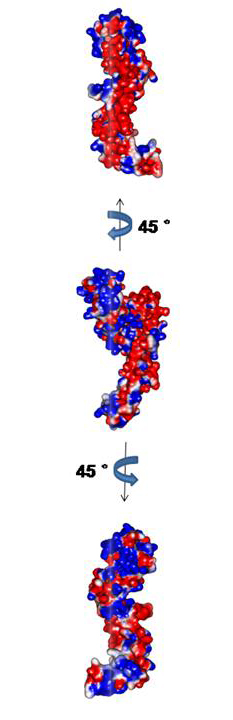


**Fig S2.** Electrostatic potential surface of m14 in the absence of EGCG. The conformation saved at 200 ns was used to calculate the electrostatic potential surface. The negative charged region (top panel) was contributed by the acidic tail. While other region on the surface has evenly-distributed charges, which is in contrast to the m17 in the presence of EGCG (Fig 3). This relative small polar surface is also agreement with the small dipole moment of 1269.4 Debye (Table 1)


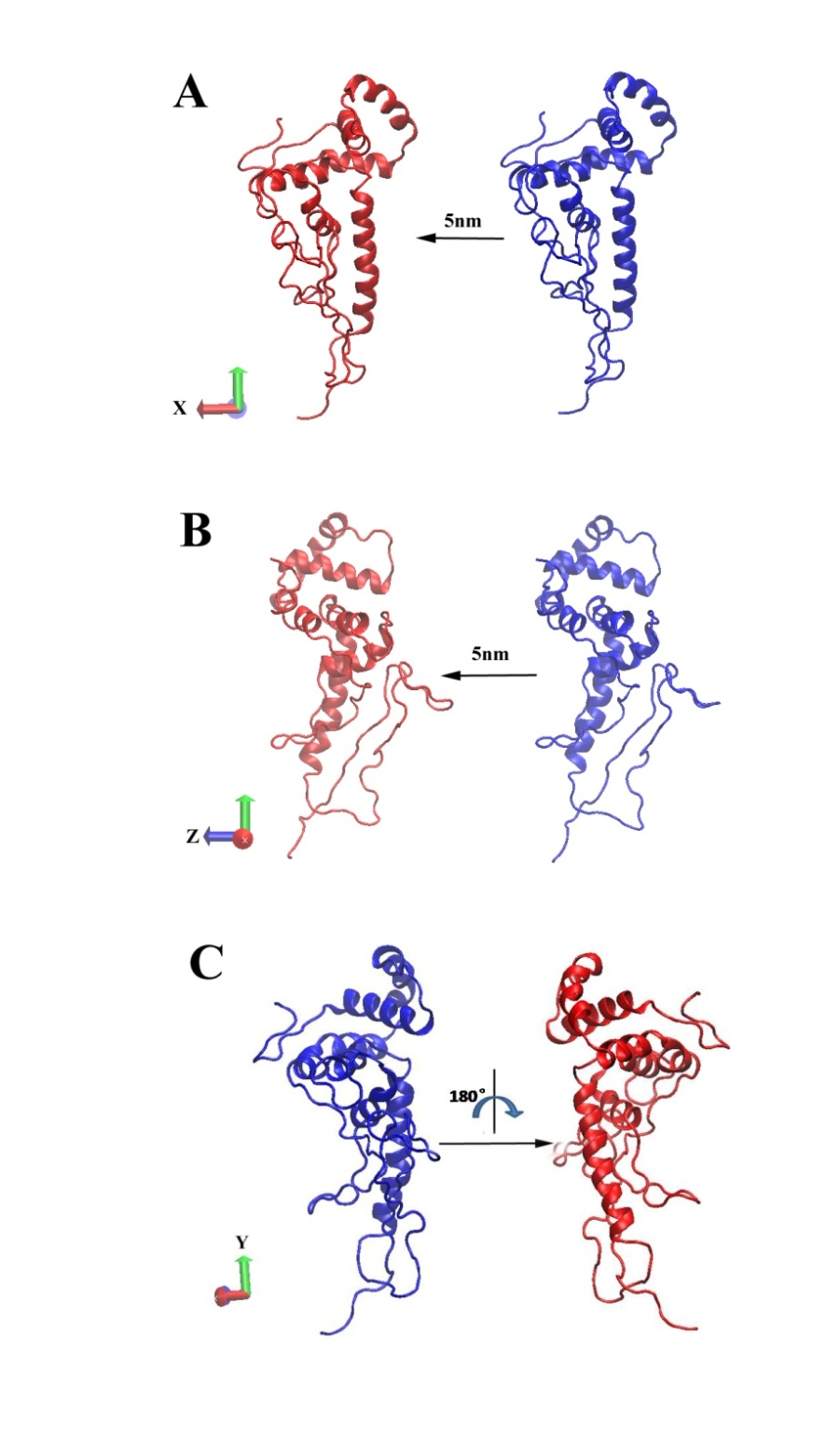


**Fig S3.** Initial configurations of m17+EGCG dimerization simulations: (A) X system (B) Z system and (C) Y system. For X and Z system, the monomer was translated 5 nm along the X and Z axis, respectively, to generate at least 1 nm distance between the monomers. For Y system, the monomer was rotated 180 degree along the Y axis followed by a translation to generate at least 1nm minimal distance between the monomers.


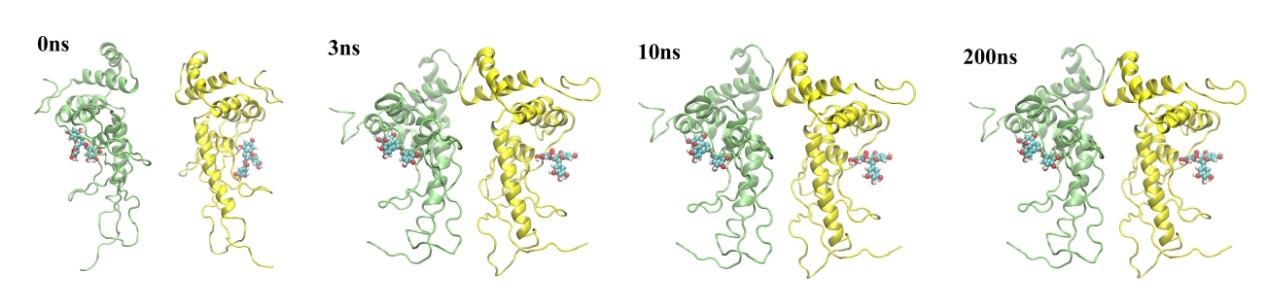


**Fig S4.** Dimerization of Y system (m17+EGCG). The relative orientation between the monomers was not changed throughout the 200 ns simulations.


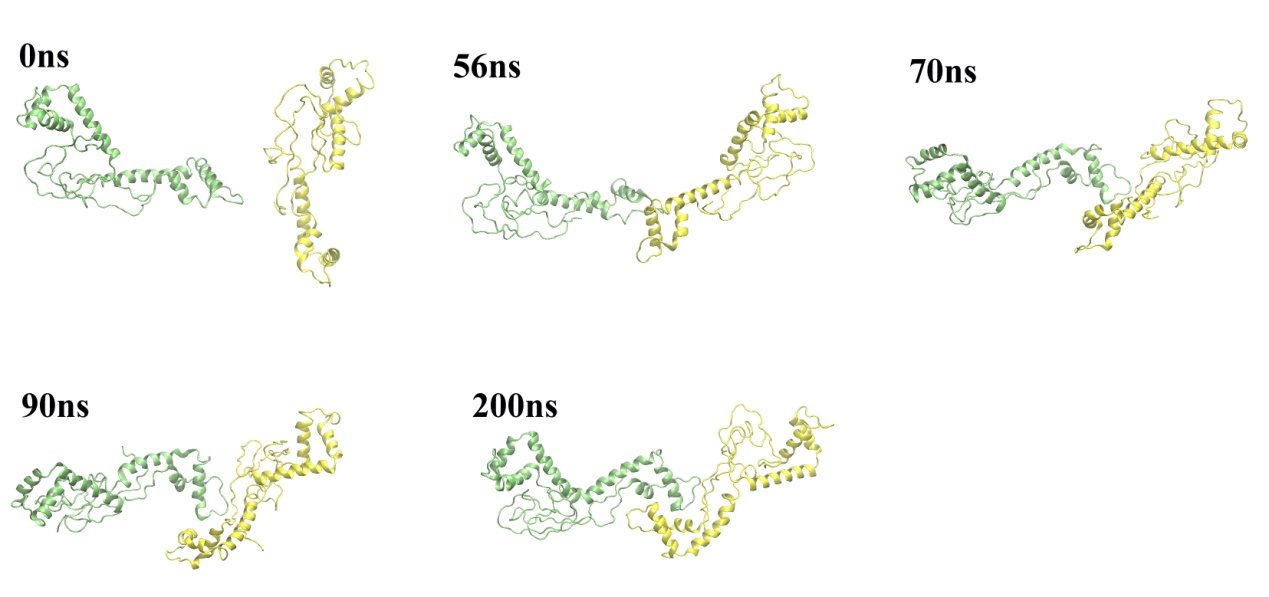


**Fig S5.** Dimerization of tail-to-AT system (m14 alone). In the initial setup (0ns), the Box B of protomer A (in green) pointed to the AT of the protomer B (in yellow). However, the Box Bs from two protomers began to interact with each other at 56ns and finally hooked up at the end of the simulation


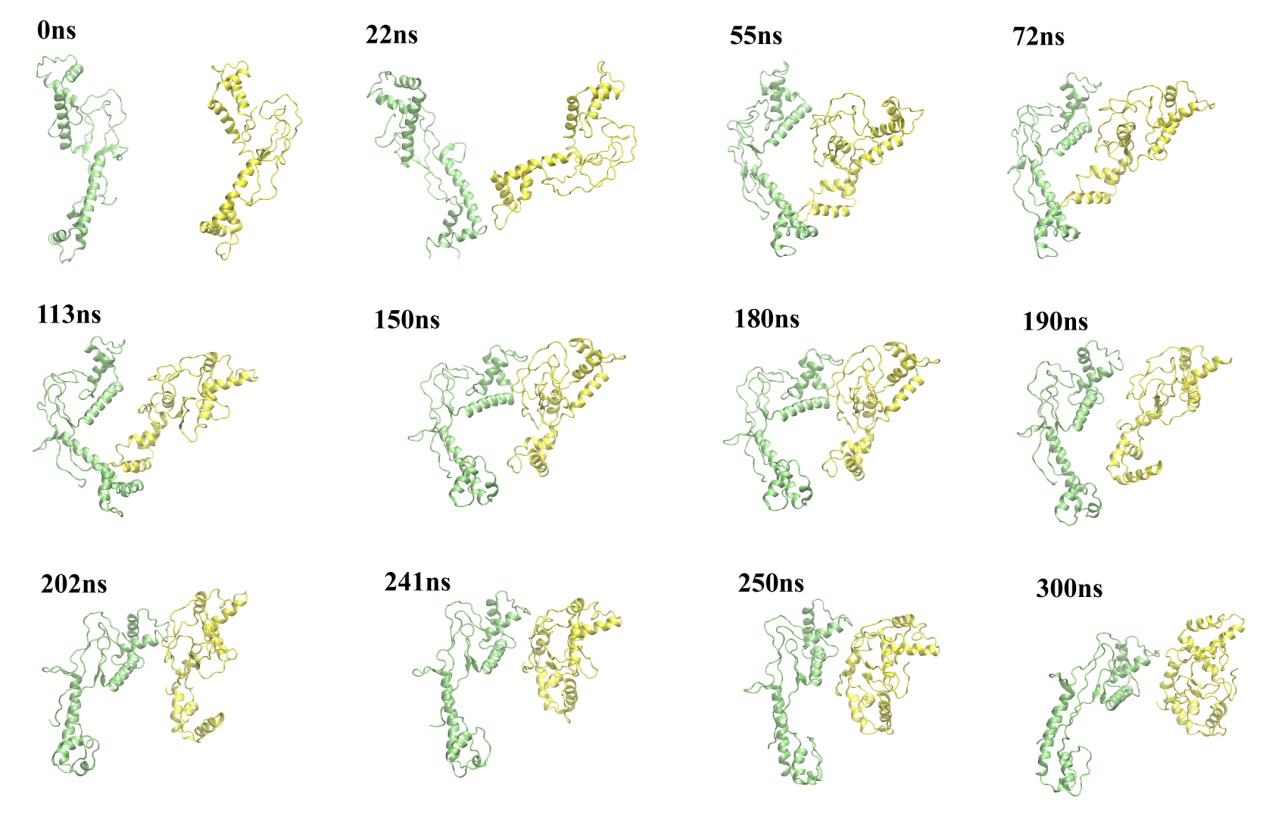


**Fig S6.** Dimerization of parallel system (m14 alone). Two protomers began to interact with other at 22 ns. However, the interface always changed even the simulation time was extended to 300 ns, which implies interaction between the protomers is unfavorable in the absence of EGCG.


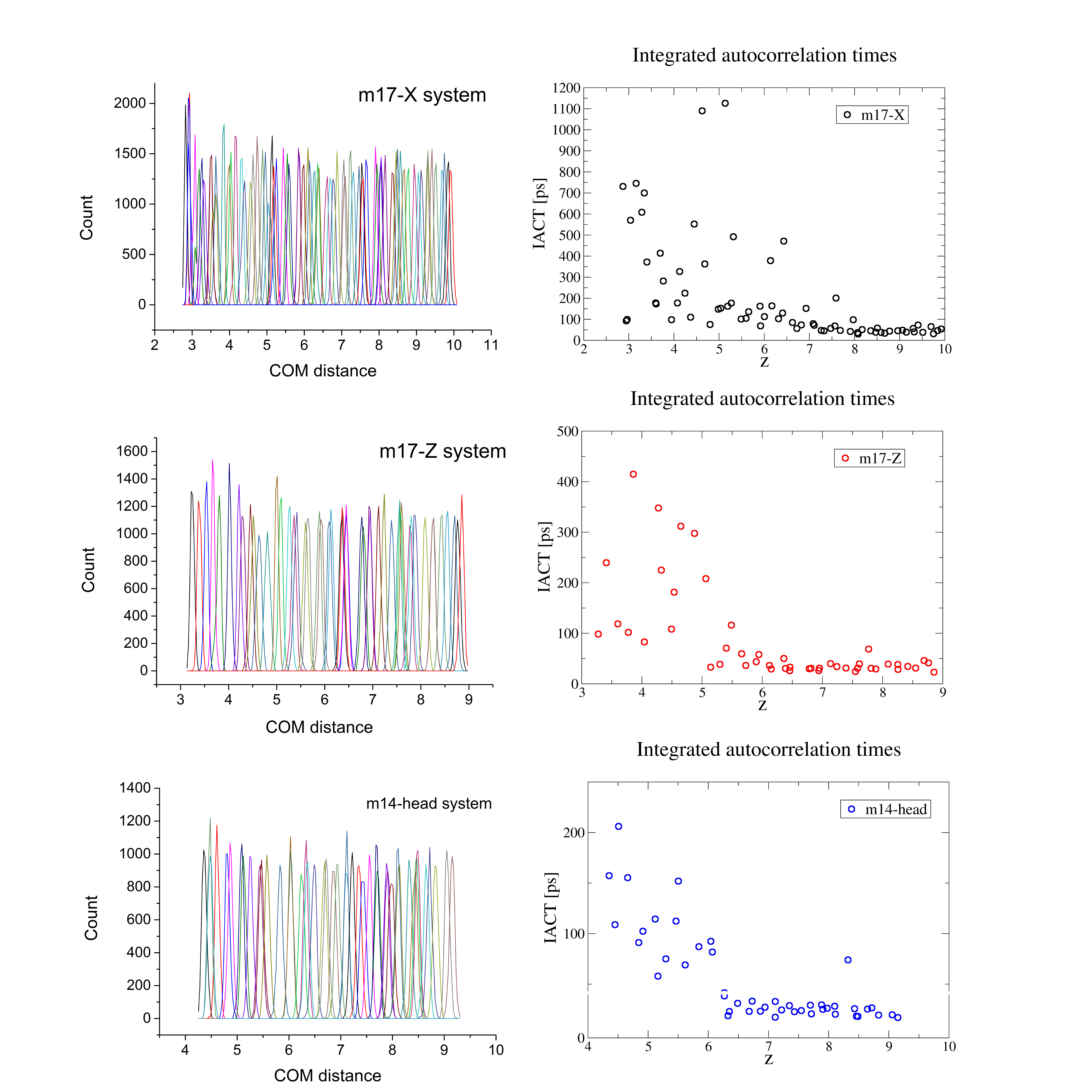


**Fig. S7.** Umbrella histograms and integrated autocorrelation times for three systems (m17 X, m17Z and m14 head) are summarized. Spacing of 0.1 nm was used to generate windows. Thus 72, 48 and 45 windows were included in the simulations of m17 X, m17 Z and m14 head systems, respectively. For each window of the umbrella sampling, the system was equilibrated for 100 ps followed by 10 ns MD simulation, which is much longer than the autocorrelation time (the longest autocorrelation time 1.1 ns).


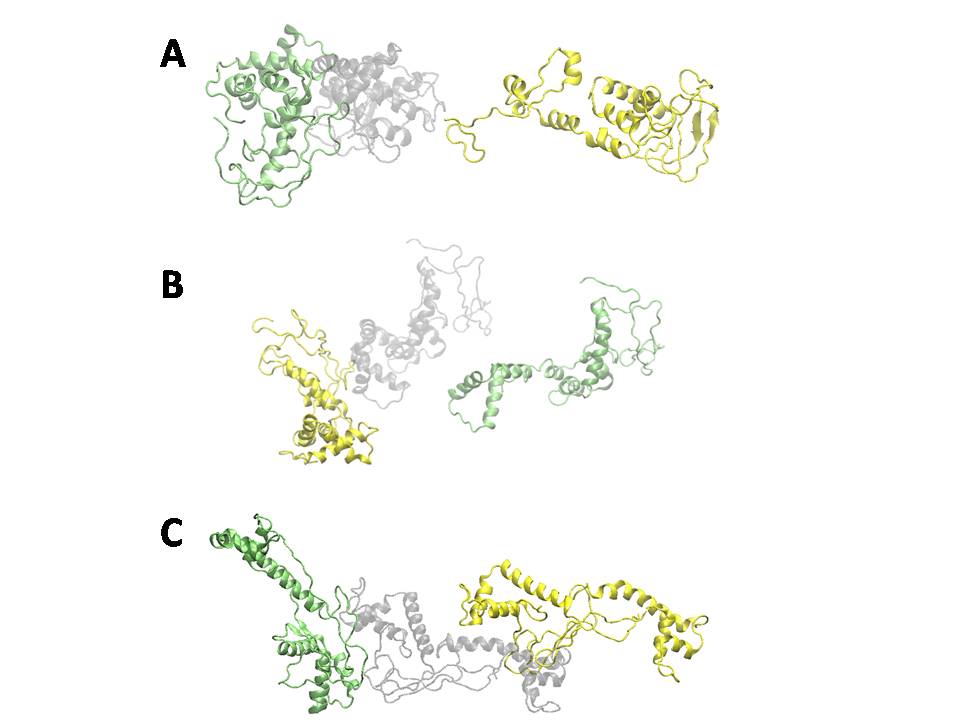


**Fig S8.** Initial and final conformations of three representative simulations in the simulations of applying pulling force:(A)X system of m17+EGCG, (B) Z system of m17+EGCG and (C)head-to-tail system of m17 without EGCG. In the three pulling simulations, conformational changes always occurred in the protomer which was applied forces.


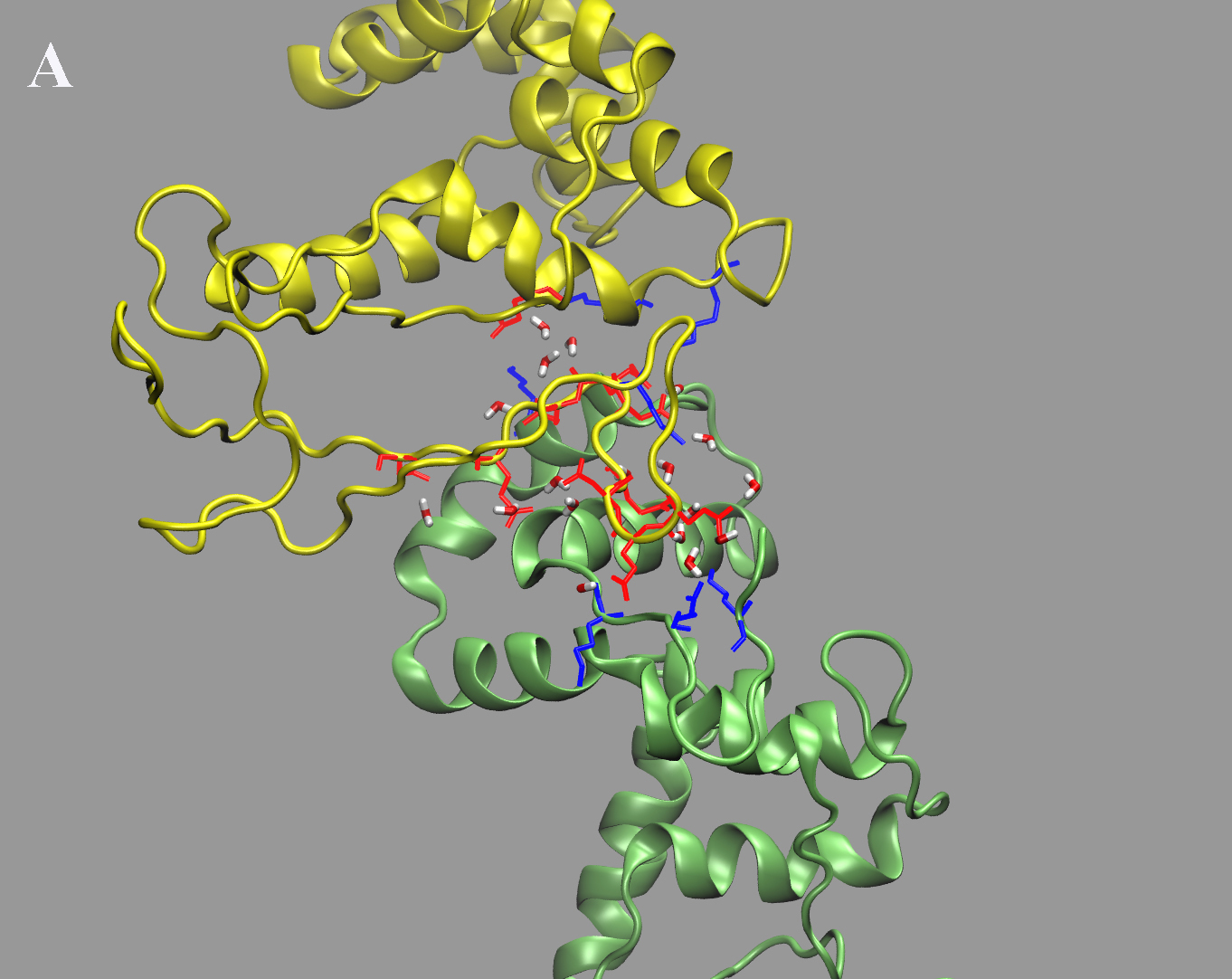


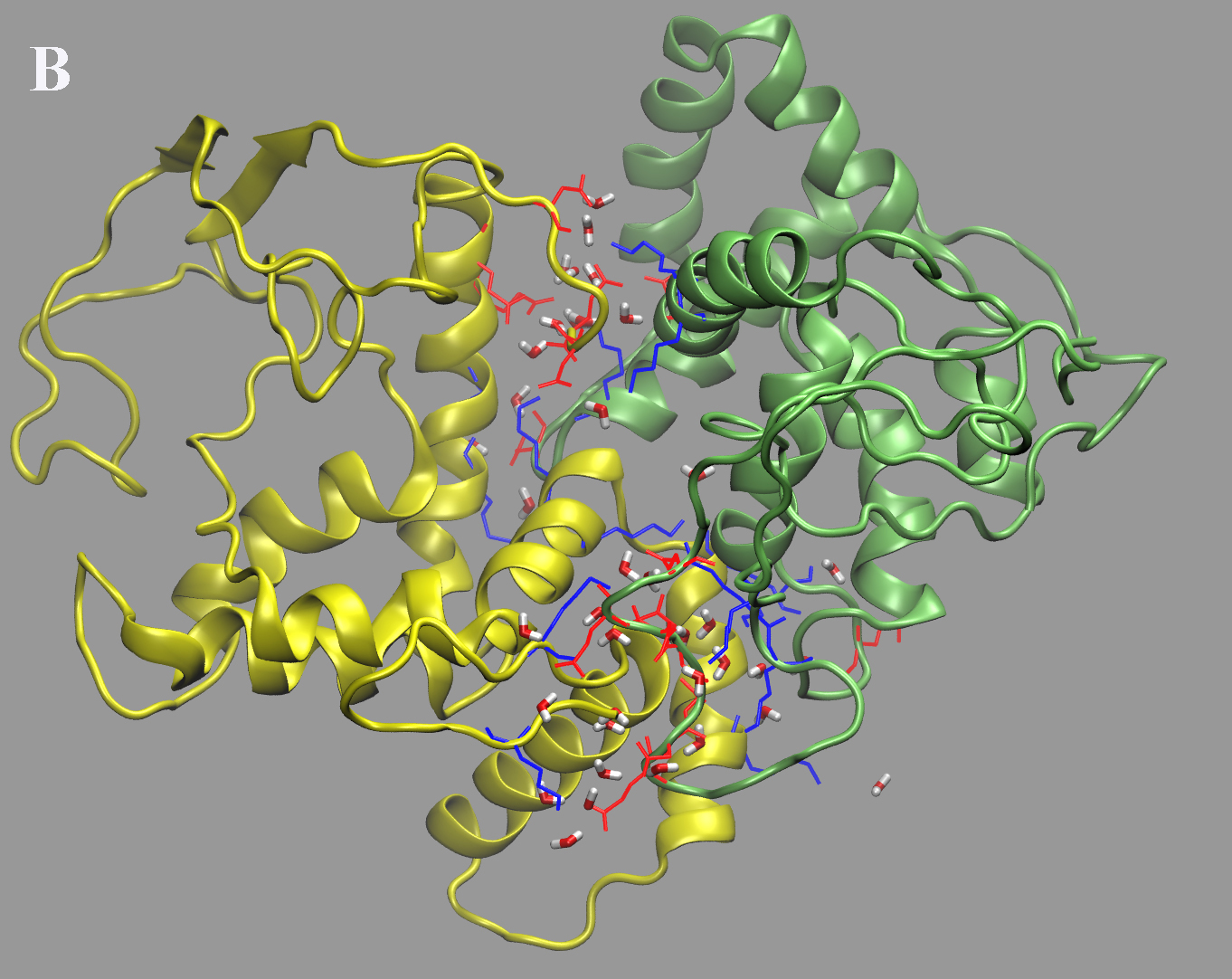


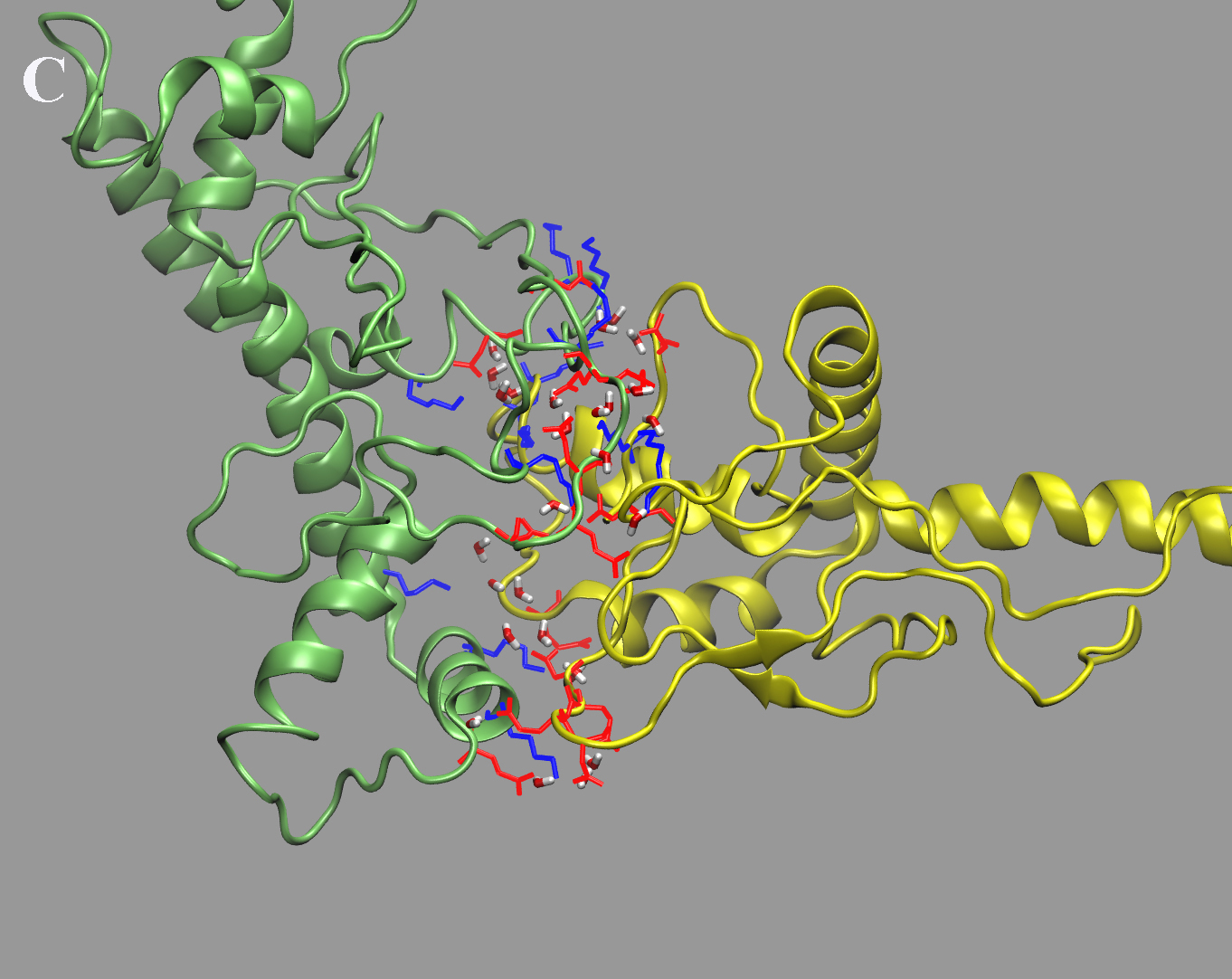


**Fig. S9.** Dimer interfaces of m17-Z (A), m17-X (B) and m14-head (C) systems. Basic residues are color in blue and acidic in red. Interface water molecules are shown in stick.
